# MitoNEET Protects HL-1 Cardiomyocytes from Oxidative Stress Mediated Apoptosis in an *In Vitro* Model of Hypoxia and Reoxygenation

**DOI:** 10.1371/journal.pone.0156054

**Published:** 2016-05-31

**Authors:** Anika Habener, Arpita Chowdhury, Frank Echtermeyer, Ralf Lichtinghagen, Gregor Theilmeier, Christine Herzog

**Affiliations:** 1 Department of Anesthesiology and Intensive Care Medicine, Hannover Medical School, Hannover, Germany; 2 Department of Pediatric Pneumology, Allergology and Neonatology, Hannover Medical School, Hannover, Germany; 3 Department of Cellular Biochemistry, University Medical Center Göttingen, Göttingen, Germany; 4 Institute for Clinical Chemistry, Hannover Medical School, Hannover, Germany; 5 Department of Health Services Sciences, Faculty of Medicine and Health Sciences, University of Oldenburg, Oldenburg, Germany; University of Catania, ITALY

## Abstract

The iron-sulfur cluster containing protein mitoNEET is known to modulate the oxidative capacity of cardiac mitochondria but its function during myocardial reperfusion injury after transient ischemia is unknown. The purpose of this study was to analyze the impact of mitoNEET on oxidative stress induced cell death and its relation to the glutathione-redox system in cardiomyocytes in an *in vitro* model of hypoxia and reoxygenation (H/R). Our results show that siRNA knockdown (KD) of mitoNEET caused an 1.9-fold increase in H/R induced apoptosis compared to H/R control while overexpression of mitoNEET caused a 53% decrease in apoptosis. Necrosis was not affected. Apoptosis of both, mitoNEET-KD and control cells was diminished to comparable levels by using the antioxidants Tiron and glutathione compound glutathione reduced ethyl ester (GSH-MEE), indicating that mitoNEET-dependent apoptosis is mediated by oxidative stress. The interplay between mitoNEET and glutathione redox system was assessed by treating cardiomyocytes with 2-acetylamino-3-[4-(2-acetylamino-2-carboxyethylsulfanylthio-carbonylamino) phenylthiocarbamoylsulfanyl] propionic acid (2-AAPA), known to effectively inhibit glutathione reductase (GSR) and to decrease the GSH/GSSG ratio. Surprisingly, inhibition of GSR-activity to 20% by 2-AAPA decreased apoptosis of control and mitoNEET-KD cells to 23% and 25% respectively, while at the same time mitoNEET-protein was increased 4-fold. This effect on mitoNEET-protein was not accessible by mitoNEET-KD but was reversed by GSH-MEE. In conclusion we show that mitoNEET protects cardiomyocytes from oxidative stress-induced apoptosis during H/R. Inhibition of GSH-recycling, GSR-activity by 2-AAPA increased mitoNEET-protein, accompanied by reduced apoptosis. Addition of GSH reversed these effects suggesting that mitoNEET can in part compensate for imbalances in the antioxidative glutathione-system and therefore could serve as a potential therapeutic approach for the oxidatively stressed myocardium.

**Highlights**

- MitoNEET protects cardiomyocytes from oxidative stress induced apoptosis
- Chemical inhibition of glutathione reductase activity by 2-AAPA reduces apoptosis and increases mitoNEET protein
- Addition of reduced glutathione reverses the effects of 2-AAPA

## Introduction

Oxidative stress is a critical factor for the enlargement of myocardial damage during reperfusion injury after transient myocardial ischemia [1–3] by inducing cardiomyocyte death through apoptosis and necrosis [4, 5]. One option to therapeutically counteract myocardial cell death is to sustain the antioxidative capacity of the myocardium.

MitoNEET is a ubiquitously expressed iron-sulfur (Fe-S) protein with putative antioxidative capacity and with the highest level of mRNA seen in the heart [6]. Initially mitoNEET was discovered as a binding partner of pioglitazone, an insulin-sensitizing drug used in type 2 diabetes. The protein is located in the outer mitochondrial membrane by a N-terminal anchor and its C-terminus is facing towards the cytoplasm [6]. According to its crystal structure mitoNEET is a homodimer with one [2Fe-2S] cluster in each monomer [7, 8]. Fe-S cluster containing proteins exhibit multiple functions depending on cluster ligands, their orientations and the local hydrogen-bonding arrangement [9]. In particular, they often take part in dynamic redox-sensitive activities; act as electron transport mediators; regulatory agents in gene expression and enzyme activity; act as a depot for sulfur as well as iron; and sensors for cellular oxygen [10–12]. In fact, in the last few years the role of mitoNEET as a redox-active protein has been described in several disease models like obesity [13], cancer [14] and inflammation-induced Parkinson`s disease [15]. However, its role in cardiovascular disease states that are associated with oxidative stress induced damage has not been characterized yet.

The glutathione (GSH) redox system is one of the main antioxidative defence systems in cardiomyocytes [4]. The relevance of glutathione-dependent processes was demonstrated by augmented ischemic tissue damage after GSH depletion [16] and transient reduction of glutathione content connected with diminished activity of the enzyme glutathione reductase (GSR) [17] that reconstitutes the antioxidative form of glutathione [18].

In this study we aim to analyze the role of mitoNEET as a putative antioxidative motif and its interaction with known antioxidative systems like the glutathione reductase system during myocardial oxidative stress. For this purpose we used an *in vitro* model of hypoxia and reoxygenation (H/R)-induced apoptosis in HL-1 cardiomyoblast-like cells [19–22]. We chose the model of H/R since chronic hypoxia alone does not cause apoptosis in cardiomyocytes in cell culture [23]. In the first step, we examined the influence of mitoNEET on oxidative stress mediated cell death. Secondly we analyzed the interplay between mitoNEET and the glutathione redox-system.

## Materials and Methods

### Cell culture

For all cell culture experiments the murine cardiomyocyte cell line HL-1 (a kind gift of Prof. W. C. Claycomb, Louisiana State University, New Orleans, LA, USA) was used. HL-1 cells are a derivative of AT-1 cardiac myocytes, derived from an atrial tumor isolated from transgenic mice, in which the expression of simian virus 40 large T antigen was controlled by the atrial natriuretic factor promotor [20, 24]. HL-1 cells were cultured as described previously [20, 25].

One day after plating HL-1 cells were transfected with 40 pmol small interfering RNA (siRNA) to mitoNEET (28929, Applied Biosystems, Darmstadt, Germany) or non-specific (scr)-siRNA (1027281, Qiagen, Hilden, Germany) using Lipofectamine RNAiMAX (Invitrogen, Karlsruhe, Germany) under serum-free conditions (Opti-MEM I Reduced Serum Medium, GlutaMAX, Gibco, Darmstadt, Germany) based on the manufacturer`s instructions. Respective siRNAs with alternative backbones delivered comparable results. 30 hours after transfection cells were washed and maintained in minimal medium (without serum and norepinephrine) over night. Cells were subjected to normoxia (5% CO_2_, 95% room air, 37°C) or simulated hypoxia (0.8% O_2_, 94.2% N_2_, 5% CO_2_, 37°C) in media that was buffered according to hypoxic or normoxic conditions to target a stable pH and pCO_2_ as described elsewhere [26]. After 3 hours cells were washed carefully and maintained in minimal medium for 1 hour during reoxygenation (H/R). Where indicated, cells were treated with GSR-inhibitor 2-acetylamino-3-[4-(2-acetylamino-2-carboxyethylsulfanylthiocarbonylamino) phenylthiocarbamoylsulfanyl] propionic acid (2-AAPA, 10 μM, dissolved in DMSO), the antioxidants Tiron (10 mM, dissolved in water) and glutathione reduced ethyl ester (GSH-MEE, 2 mM, dissolved in water, all Sigma-Aldrich, Munich, Germany) 30–60 minutes before and during H/R.

### Protein overexpression

Mouse mitoNEET cDNA (genbank accession number NM_134007) was PCR amplified from position 96 to 422 with primer 5’-AC GCTAGC T GACT ATG GGC CTC AGC TCC AAC T-3’ and 5’-AC GAATTC AGC TTA GGT TTC CTT TTT CTT GAT GA-3’ using mouse HL-1 cDNA and subcloned into the expression vector pcDNA3.1 (Invitrogen, Karlsruhe, Germany). One day after plating HL-1 cells were transfected with 1 μg pcDNA3.1 or mitoN-pcDNA3.1 using X-tremeGENE^™^ 9 Transfection Reagent (Sigma-Aldrich, Munich, Germany) based on the manufacturer`s instructions. 30 hours after transfection cells were maintained in minimal medium overnight and subjected to H/R as described before.

### Real-time RT-PCR

Total RNA was extracted from HL-1 cells using TRItidy reagent (Applichem, Darmstadt, Germany) and measured photometrically at 260/280 nm. Reagents and primers were obtained from Eurogentec (Cologne, Germany). 500 ng of total RNA was applied for reverse transcription using Reverse Transcriptase Core kit according to the manufacturer`s protocol. cDNA was diluted 1:1 in DNase-, RNase- and protease-free water (Applichem, Darmstadt, Germany) and 0.5 μl template was used.

Sequences of primer-pairs were as follows: mouse mitoNEET forward, 5’-CGCTAAAGAGAATCGCACCAAAGC-3’, and mitoNEET reverse, 5’-GCCTCGCAACTGTCCATTAGGTTT-3’; mouse HPRT forward, 5’-TGATCAGTCAACGGGGGACATA-3’, and HPRT reverse, 5’-GCCTGTATCCAACACTTCGAGA-3’. For real-time RT-PCR MESA-GREEN qPCR MasterMix Plus for SYBR Assay was used based on the manufacturer`s procedure. Signals generated by integration of MESA-GREEN into the amplified DNA were detected in a real-time machine (Rotogene 3000, Corbett Life Science, Hilden, Germany) and normalized to hypoxanthine phosphoribosyltransferase (HPRT) gene expression. Data are expressed as 2^-ΔΔCT^.

### Lactate dehydrogenase (LDH)

Cell culture supernatants were harvested after H/R and LDH concentrations were measured as U/L using the LDH IFCC kit (Modular P, Roche Diagnostics, Mannheim, Germany) by a routine clinical analyzer (Hitachi 917, Hitachi Ltd, Tokyo, Japan). Data are expressed as % of hypoxic control.

### Generation of polyclonal mitoNEET antibodies

Rabbit polyclonal antibodies specific for mitoNEET were generated by 28-day protocol using a double peptide strategy (Eurogentec, Seraing, Belgium). Two separate peptide sequences (EP083398: H2N-VHAFDMEDLGDKA+C-CONH2 and EP083397: H2N-C+LAYKKFYAKENRTKA-CONH2) corresponding to mouse mitoNEET-protein (UniProt ID: Q91WS0) were synthesized and boosted by keyhole limpet hemocyanin, a non-Freunds adjuvant. Two rabbits were immunized 4 times with both mitoNEET peptides. Polyclonal anti-sera of both hosts were affinity purified against the injected peptides respectively. Specificity of the polyclonal antibodies was verified by siRNA transfection and competition experiments by Western Blot as well as mitochondrial localization by immunofluorescence microscopy. Due to its higher specifity the polyclonal antibody against peptide EP083398 of rabbit SY1094 was chosen for further studies (see S1 Protocol and S1 Fig).

### Western Blot analysis

HL-1 cells were harvested with RIPA lysis buffer (50 mM Tris-HCl pH 7.4, 150 mM NaCl, 1% Nonidet P-40, 0.1% SDS, 0.5% Sodiumdesoxycholate) supplemented with phosphatase and protease inhibitors (PhosStop and Complete Mini, Roche Applied Science, Mannheim, Germany) and protein concentration of the supernatants were determined by Pierce BCA Protein Assay Kit (Thermo Scientific, Karlsruhe, Germany). Protein lysates (0,6–10 μg) were separated on 4–20% Tris-glycine gels (PAGEr Precast Gels, Lonza, Cologne, Germany) under reducing conditions. Proteins were blotted on PVDF membranes (Milipore, Darmstadt, Germany) for 1 hour which were then blocked with 5% nonfat dry milk in Tris buffered saline supplemented with 0.05% Tween20 (TBST 0.05%), 4% BSA/TBST 0.05% or 4% IgG-free, protease-free BSA/TBST 0.1% for 1 hour at room temperature (RT). Membranes were incubated with the following primary antibodies over night at 4°C: mitoNEET (diluted 1:20000 in 4% BSA/TBST 0.05%); glutathione reductase (ABIN233969, Antibodies Online, Aachen, Germany, diluted 1:5000 in 5% nonfat dry milk/TBST 0.05%), cleaved caspase-3 (no. 9661, Cell Signaling, Frankfurt, Germany, diluted 1:2000 in 4% IgG-free, protease-free BSA/TBST 0.1%). Horseradish-peroxidase-coupled rabbit IgG was used as secondary antibody (R1364HRP, Acris, Herford, Germany or P0448, Dako, Hamburg, Germany, diluted 1:2000–1:20000 in BSA or nonfat dry milk/TBST). The blots were detected on a ChemiSmart 5100 station (Vilbert Lourmat, Eberhardzell, Germany) by using Amersham ECL Plus Western Blotting Detection System (GE Healthcare, Braunschweig, Germany). Density of bands were quantified using Bio1D software (Vilber Lourmat, Eberhardzell, Germany). To correct signals for equal protein loading, membranes were re-probed with anti-Glyceraldehyde-3-Phosphate Dehydrogenase (GAPDH) (no. 2118, diluted 1:2000), anti-β-tubulin (no. 2146, diluted 1:1000–1:5000) or anti-β-actin (no. 4967, diluted 1:5000, all Cell Signaling, Frankfurt, Germany). Data are expressed as % of H/R control.

### Apoptosis-Assays

Apoptosis was assessed by dectection of cleaved caspase-3 in Western Blot (see Western Blot analysis) and by TdT-mediated dUTP-biotin nick end labeling (TUNEL) method using the MEBSTAIN Apoptosis Kit II according to the manufacturer’s instructions (MBL,Woburn, MA, USA).

### Measurement of intracellular Reactive Oxygen Species (ROS) in HL-1 cells

Oxidative radicals generated in HL-1 cells were measured by using 2’,7’-dichlorofluorescin diacetate (DCF-DA; Sigma-Aldrich, Munich, Germany). DCF-DA is an cell permeable probe, which is de-esterified intracellularly and becomes fluorescent after oxidation. 7 x 10^4^ HL-1 cells were pre-incubated with 5 μM DCFH-DA in hypoxia-medium (see cell culture) for 15 min at 37°C. The fluorescence intensitiy was measured before hypoxia, after 3h hypoxia and after 3h hypoxia and 60 min reoxygenation in 12 well plates using a microplate reader (Spectrafluor Plus; Tecan, Crailsheim, Germany).

### Glutathione reductase enzyme activity

Glutathione reductase (GSR)-activity was measured by a colorimetric glutathione reductase assay (no. K71-61-200, BioVision, Mountain View, CA, USA) where GSR reduces GSSG to GSH, which reacts with 5, 5′-Dithiobis (2-nitrobenzoic acid) (DTNB) to generate TNB^2-^ (yellow color, λ max = 405 nm). Samples and standards were detected at 405 nm in a microplate reader (Sunrise, Tecan, Crailshaim, Germany). The activity was calculated, normalized to protein amount (mU/mg protein) and expressed as % of H/R control.

### Statistical analysis

For all statistical analysis GraphPad Prism software Version 5.0d was used (GraphPad Inc, San Diego, CA, USA). Data are presented as mean ± standard error of mean. Whenever data showed non-Gaussian distributions or significantly different standard deviations, Kruskal-Wallis test was used followed by Mann-Whitney-U test. Otherwise ANOVA was chosen followed by t-test.

## Results

### MitoNEET plays a role for oxidative stress induced apoptosis in cardiac HL-1 cells

**Fig 1:**
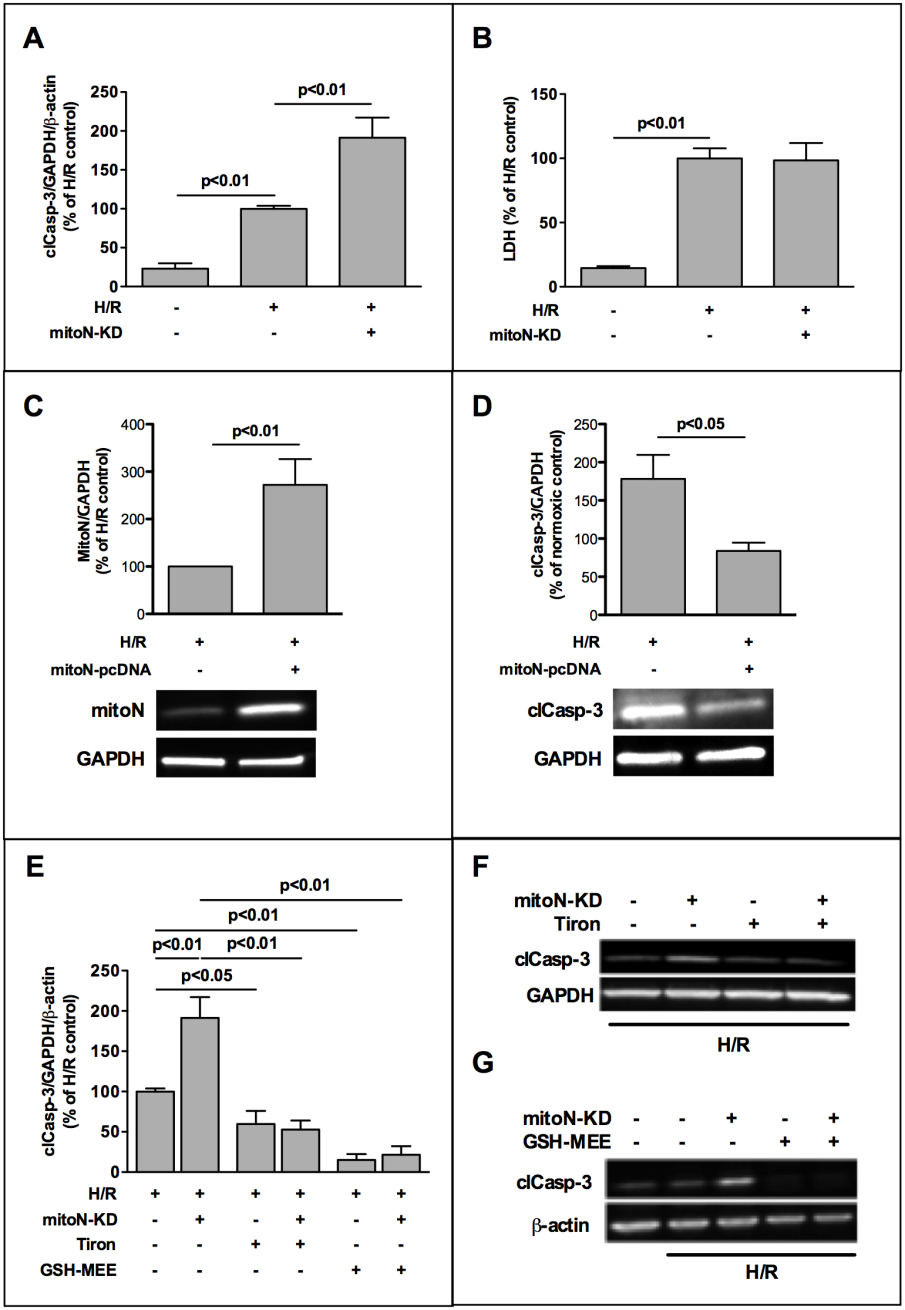
MitoNEET plays a role for oxidative stress induced apoptosis in cardiac HL-1 cells. HL-1 cells were transfected with silencing RNA (siRNA) directed against mitoNEET and non-specific-siRNA as control and subjected to 3h hypoxia followed by 1h of reoxygenation (H/R). (A) Densitometric analysis of Western Blots revealed aggravated activation of caspase-3 in mitoNEET-knockdown (mitoN-KD) cells after H/R compared to hypoxic controls (n = 11). (B) MitoNEET-KD showed no effect on lactate dehydrogenase (LDH) release after H/R measured in culture supernatants (n = 10). LDH was quantified as U/L by a routine clinical analyzer and is expressed as % of H/R control. (C,D) Overexpression of mitoNEET in HL-1 cells caused a significant decrease in apoptosis in mitoNEET overexpressing cells compared to H/R control cells. Expression of mitoNEET (n = 5) and cleaved caspase (n = 7) is shown in representative Western Blots and was densitometrically measured as % of H/R control. (E-G) H/R-induced apoptosis in control- and mitoNEET-KD cells was reduced by two different antioxidants, superoxide scavenger Tiron (10 mM, n = 5) and esterified glutathione compound GSH-MEE (glutathione reduced ethyl ester, 2 mM, n = 6) as demonstrated by representative Western Blots (F-G). Data were analyzed densitometrically, normalized to housekeeping gene expression and are expressed as % of H/R control.

In HL-1 cells knockdown of mitoNEET (mitoNEET-KD) expression by siRNA reduced both mitoNEET-mRNA and -protein to 25% quantified by real-time RT-PCR and Western Blot, respectively (S2 Fig). We observed an increase of 1.9 fold in caspase-3 activity in the mitoNEET-KD cells after H/R compared to hypoxic control cells (Fig 1A, 1E and 1F). However, no effect was observed on necrosis measured by LDH release (Fig 1B). This demonstrated that mitoNEET-KD elevates apoptosis but not necrosis. In order to prove a direct cytoprotective effect of mitoNEET we overexpressed mitoNEET in HL-1 cells (Fig 1C) and observed a 53% reduction of active caspase-3 after H/R in mitoNEET overexpressing cells compared to hypoxic control cells (Fig 1D and S3 Fig). Addition of antioxidative superoxide scavenger Tiron (10 mM) decreased H/R-induced apoptosis of control cells to 60% (Fig 1E and 1F) as well as apoptosis of mitoNEET-KD cells to the same level indicating that mitoNEET-counteracted apoptosis was caused by superoxide radicals and was consequently related to reactive oxygen species (ROS). To assess the role of glutathione redox system in our H/R model, we used glutathione reduced ethyl ester (GSH-MEE) (Fig 1E and 1G), an esterified glutathione compound that is readily transported into the cell and increases intracellularly the bioavailability of glutathione [27]. GSH-MEE reduced apoptosis of both, hypoxic control- and mitoNEET-KD cells to the level of normoxic cells.

### MitoNEET has no impact on H/R induced intracellular ROS-production

**Fig 2:**
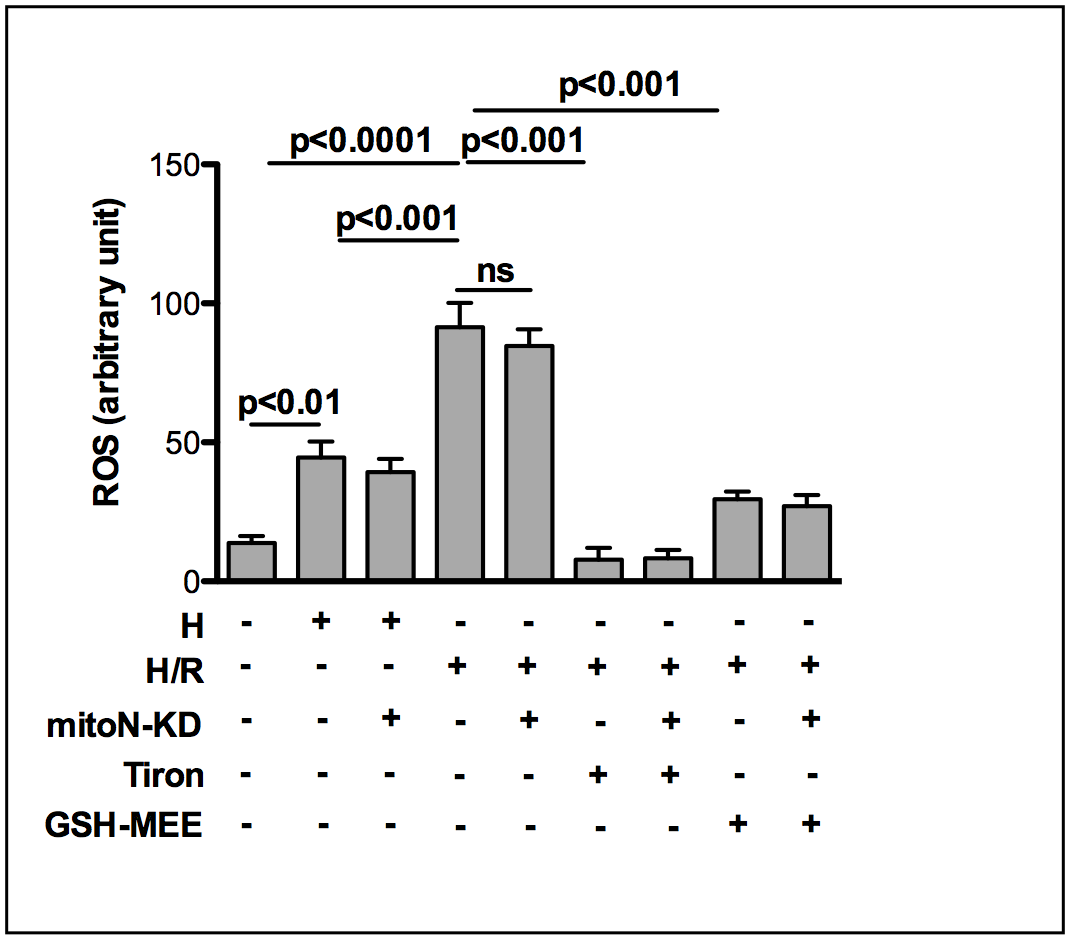
Intracellular ROS-production in HL-1 cells is not directly affected by mitoNEET. HL-1 cells were transfected with silencing RNA (siRNA) directed against mitoNEET and non-specific-siRNA as control and subjected to 3h hypoxia followed by 1h of reoxygenation (H/R). Using the fluorescent probe DCF-DA intracellular ROS-production was analyzed in a microplate reader. After 3h hypoxia intracellular ROS is 2.6 fold and after 60 min of reoxygenation even 5.4 fold increased compared to normoxic control cells (n = 12). Addition of antioxidative superoxide scavenger Tiron (10 mM; n = 6) and GSH-MEE (2 mM; n = 9) reduced the amount of ROS significantly. MitoNEET-KD doesn’t show any effect on the amount of intracellular ROS measured within the different conditions.

To analyze whether ROS play a role in our *in vitro* H/R-model we used the fluorescence probe DCF-DA to measure intracellular ROS. Our data show that after 3h hypoxia intracellular ROS is 2.6 fold and after 3h/1h H/R hypoxia even 5.4 fold increased compared to normoxic control cells (Fig 2). Addition of antioxidative superoxide scavenger Tiron (10 mM) decreased H/R-induced ROS-production by 91% and GSH-MEE (2 mM) by 70%, respectively. However, mitoNEET-KD doesn’t show any effect on the amount of intracellular ROS measured within this cell culture system.

### Chemical inhibition of glutathione reductase (GSR)-activity by 2-AAPA reduces oxidative stress induced apoptosis

Based on the literature we know that GSR-activity is decreased in rodent and human myocardial infarction and myocardial ischemia with reperfusion [28–30]. To analyze the role of GSR-activity in our H/R model we used 2-AAPA as an chemical inhibitor to effectively knockdown GSR-activity and to diminish the GSH/GSSG ratio causing an accumulation of GSSG [31–34].

**Fig 3:**
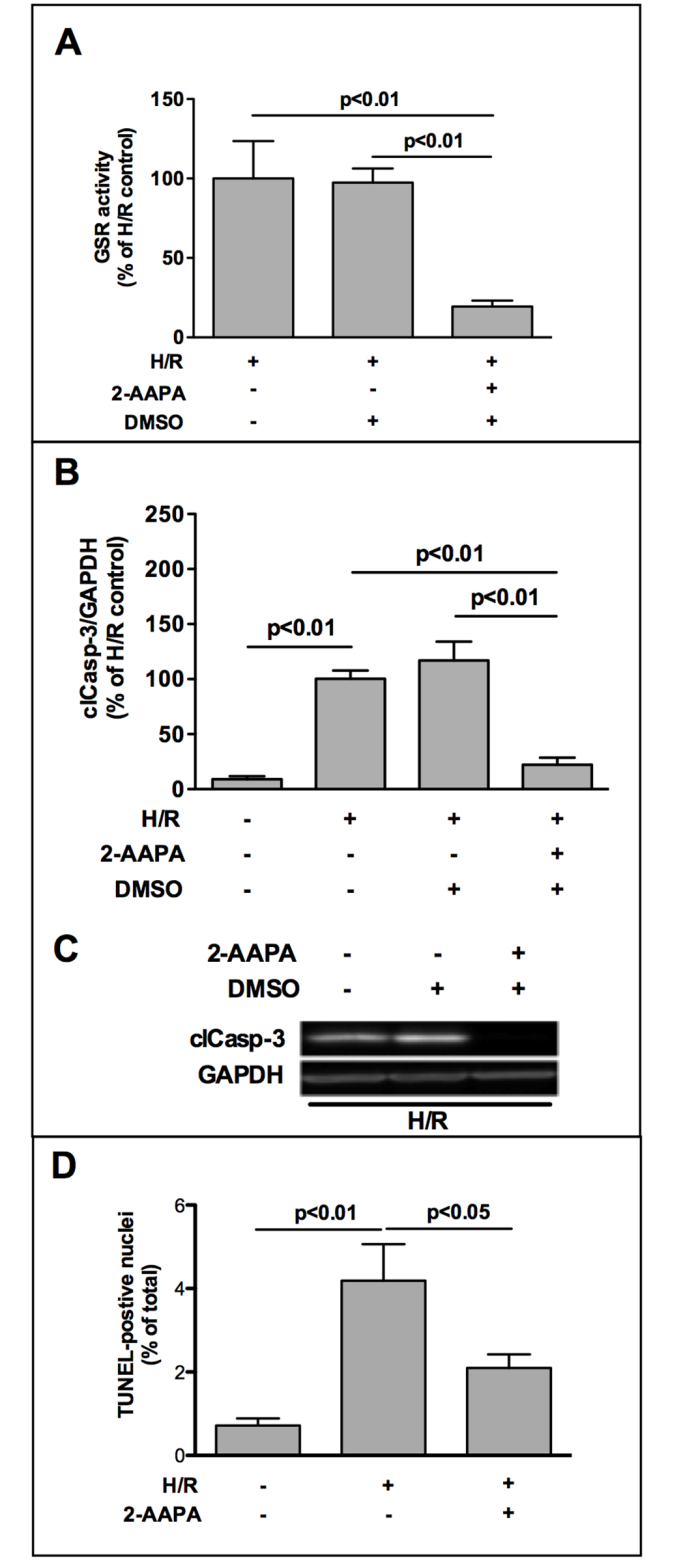
Chemical inhibition of glutathione reductase (GSR) reduces oxidative stress-induced apoptosis. (A) Chemical GSR-inhibitor 2-AAPA (10 μM) reduced GSR-activity to 20% (n = 5). GSR-activity was determined by a colorimetric glutathione reductase assay. Thereby GSR reduces GSSG to GSH that reacts with 5, 5′-Dithiobis (2-nitrobenzoic acid) (DTNB) to generate yellow TNB^2-^, which was measured at 405 nm in a microplate reader. GSR-activity was calculated, normalized to protein amount (mU/mg protein) and is expressed as % of H/R control. (B) Blockade of GSR-activity by 10 μM 2-AAPA decreased levels of activated caspase-3 in HL-1 cells after H/R (n = 5). (C) Representative Western Blot image is shown. Data were densitometrically analyzed and are expressed as % of H/R control. (D) TUNEL staining was utilized to determine apoptosis in HL-1 cells by the use of MEBSTAIN Apoptosis Kit II according to manufacturer`s protocol (MBL, Woburn, MA, USA). Data are presented as TUNEL-positive nuclei per total nuclei (n = 5).

Treatment of HL-1 cells with 10 μM 2-AAPA reduced GSR-activity to 20% (Fig 3A) without affecting GSR-protein expression (S4 Fig).

Inhibition of GSR-activity by 2-AAPA decreased apoptosis to 22% compared to H/R control (Fig 3B and 3C). To confirm this effect of 2-AAPA on H/R induced apoptotic cell death, we used TdT-mediated dUTP-biotin nick end labeling (TUNEL) method to visualize DNA fragmentation and observed a reduced amount of TUNEL-positive nuclei in 2-AAPA treated cardiomyocytes compared to H/R control cells (Fig 3D).

### MitoNEET is increased by 2-AAPA

**Fig 4:**
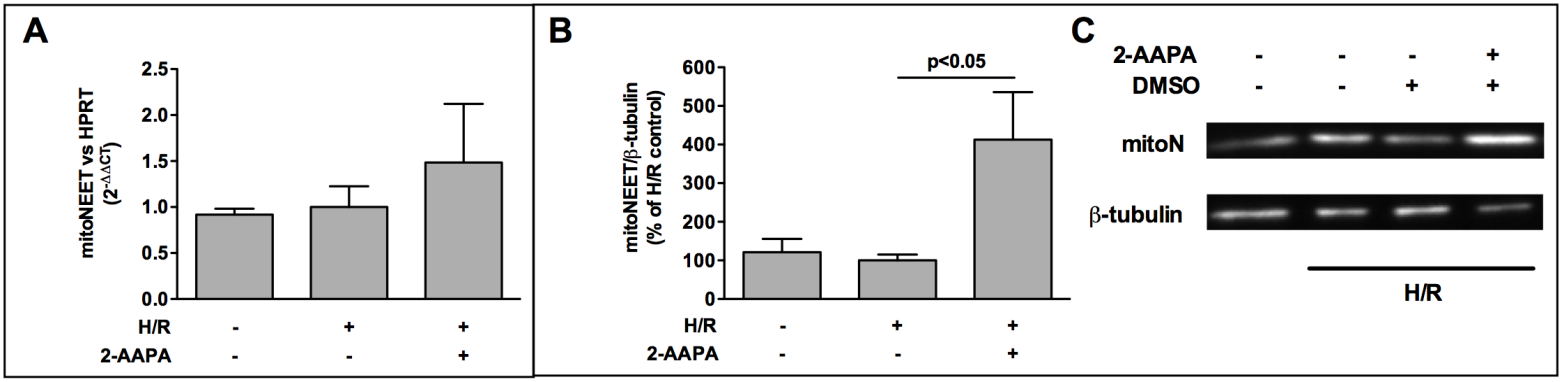
MitoNEET is increased by 2-AAPA. (A) Addition of GSR-inhibitor 2-AAPA (10 μM) led to a mild increase of mitoNEET-mRNA (n = 5) (B) while protein was increased 4-fold (n = 6) after H/R. (C) Representative Western Blots is presented. Data are expressed as % of H/R control. Real-time RT-PCR signals were normalized to hypoxanthine phosphoribosyl-transferase (HPRT) gene expression and data are expressed as 2^-ΔΔCT^.

To elucidate the underlying mechanism that governs the surprising effect of 2-AAPA on apoptosis we analyzed the impact of 2-AAPA treatment on mitoNEET expression after H/R. While mitoNEET expression is not different at mRNA- and protein-level in normoxic- compared to H/R treated HL-1 cells (Fig 4A–4C), 2-AAPA results in a mild, but not significant, elevation of mitoNEET-mRNA (Fig 4A) and a robust 4-fold increase in mitoNEET-protein (Fig 4B and 4C).

### 2-AAPA decreases H/R-induced apoptosis and increases mitoNEET-protein despite of mitoNEET-knockdown

**Fig 5:**
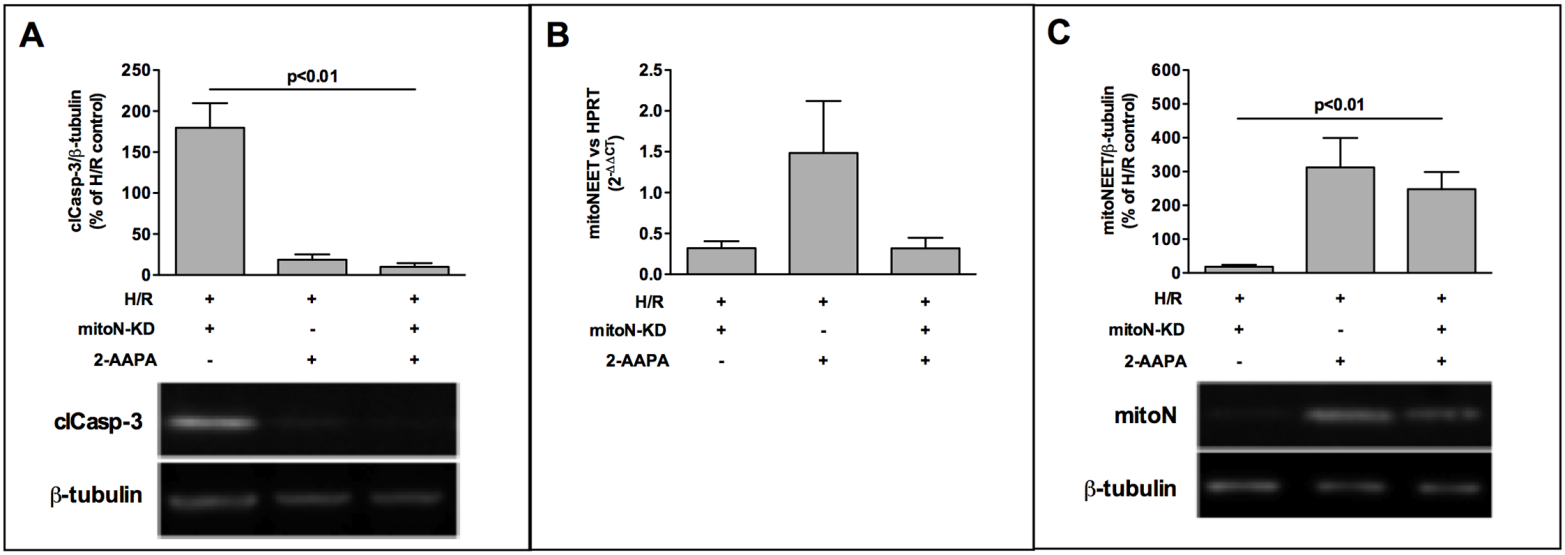
2-AAPA decreases H/R induced apoptosis in mitoNEET-KD cells and increases mitoNEET-protein despite KD. (A) Western Blot analysis of activated caspase-3 revealed reduced apoptosis in 2-AAPA treated mitoNEET-KD cells to the same level seen in 2-AAPA treated HL-1 cells after H/R (n = 6). (B) siRNA transfection decreased mitoNEET-mRNA efficiently in the presence and absence of chemical GSR-inhibitor 2-AAPA (10 μM, n = 5) determined by real-time RT-PCR. (C) MitoNEET-protein of mitoNEET-KD-cells was significantly higher after addition of 2-AAPA compared to mitoNEET-KD-cells without 2-AAPA treatment (n = 5) measured after H/R. Representative Western Blots are shown (A, C) and data are expressed as % of H/R control. Real-time RT-PCR data are expressed as 2^-ΔΔCT^.

Our data show that knockdown of mitoNEET aggravates cardiac apoptosis during H/R and GSR-inhibition by 2-AAPA increases mitoNEET amount along with decreased apoptosis. Based on these findings we were interested in the effect of 2-AAPA on apoptosis in mitoNEET-KD cells. The combination of 2-AAPA with mitoNEET-KD reversed the proapoptotic effect of mitoNEET-KD and resulted in a significant decrease of cleaved caspase-3 to a similar level as observed in H/R cells treated with 2-AAPA only (Fig 5A). On mRNA level siRNA treatment effectively reduced mitoNEET-mRNA level in the presence of 2-AAPA in H/R treated KD-cells (Fig 5B). In mitoNEET-KD-cells treated with 2-AAPA mitoNEET-protein was significantly higher than in mitoNEET-KD-cells without 2-AAPA and comparable to increased mitoNEET-protein in H/R cells treated with 2-AAPA (Fig 5C).

### Effect of 2-AAPA on mitoNEET can be reversed by GSH

**Fig 6:**
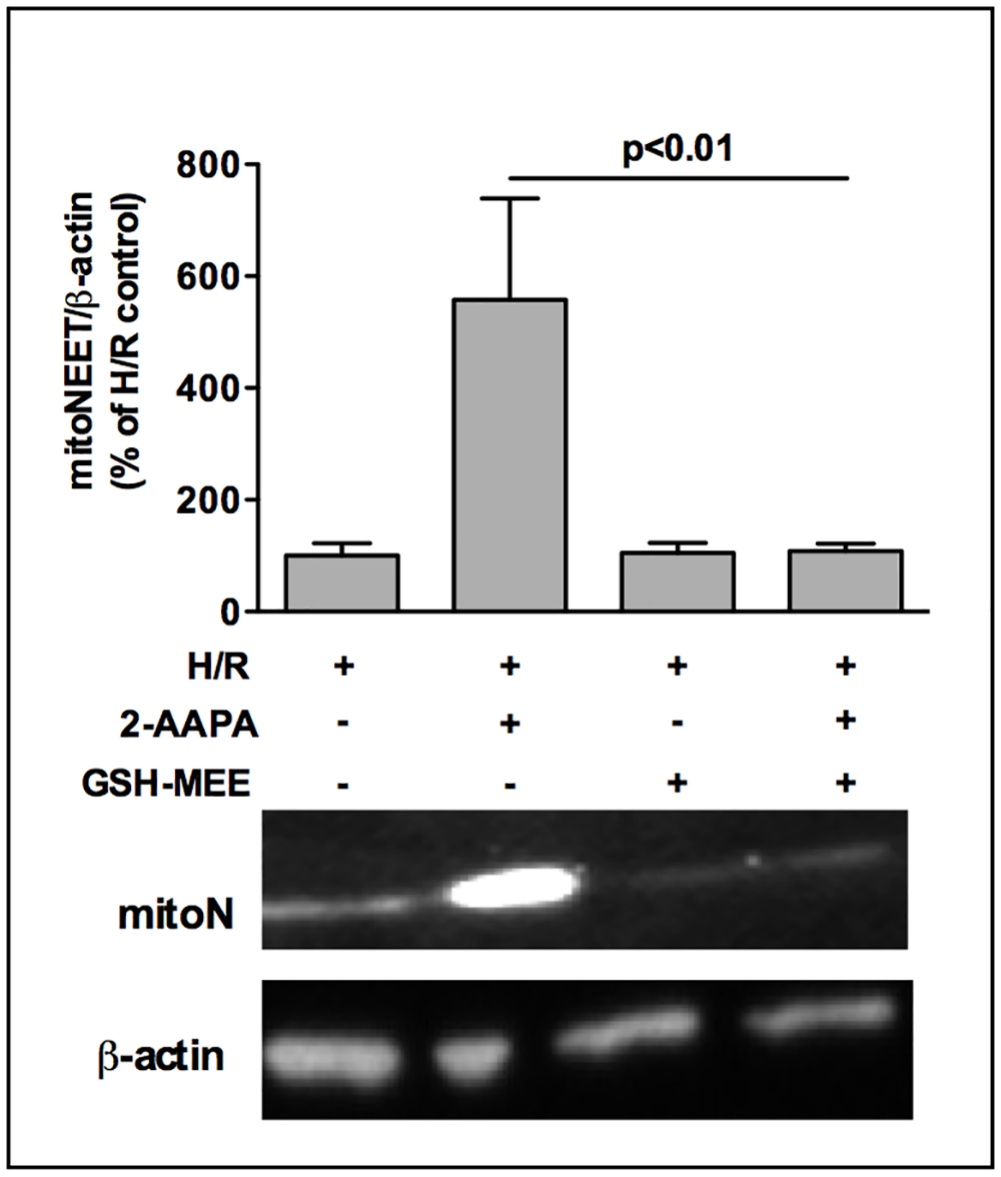
2-AAPA effect on mitoNEET is abolished by addition of glutathione compound GSH-MEE. MitoNEET-protein amount of HL-1 cells was clearly increased after addition of GSR-inhibitor 2-AAPA (10 μM) determined after H/R by Western Blot. This effect was completely reversed by additon of glutathione compound GSH-MEE (2 mM, n = 4–5) as demonstrated by a representative Western Blot. Data are expressed as % of H/R control.

To examine whether the effect of 2-AAPA on mitoNEET-protein was directly related to inhibition of GSR-activity and subsequently to GSH/GSSG ratio we added 2 mM GSH-MEE to H/R treated HL-1 cells which completely blocked the 2-AAPA induced mitoNEET increase (Fig 6) indicating that mitoNEET-protein is regulated by the redox state of the glutathione system. To address the question whether treatment of HL-1 cells with 2-AAPA and GSH-MEE shows an effect on the localization of mitoNEET within the cells we performed double immunofluorescence stainings using the mitochondrion-selective dye MitoTracker (100 nM) and rabbit anti-mitoNEET antibody (1:500). Using a 60x objective together with a 1.6x magnification changer we observed a mitochondrial colocalization of mitoNEET with MitoTracker in normoxic as well as in H/R-treated HL-1 cells, which was not affected by treatment of cells with 2-AAPA and GSH-MEE (S5 Fig).

## Discussion

**Fig 7:**
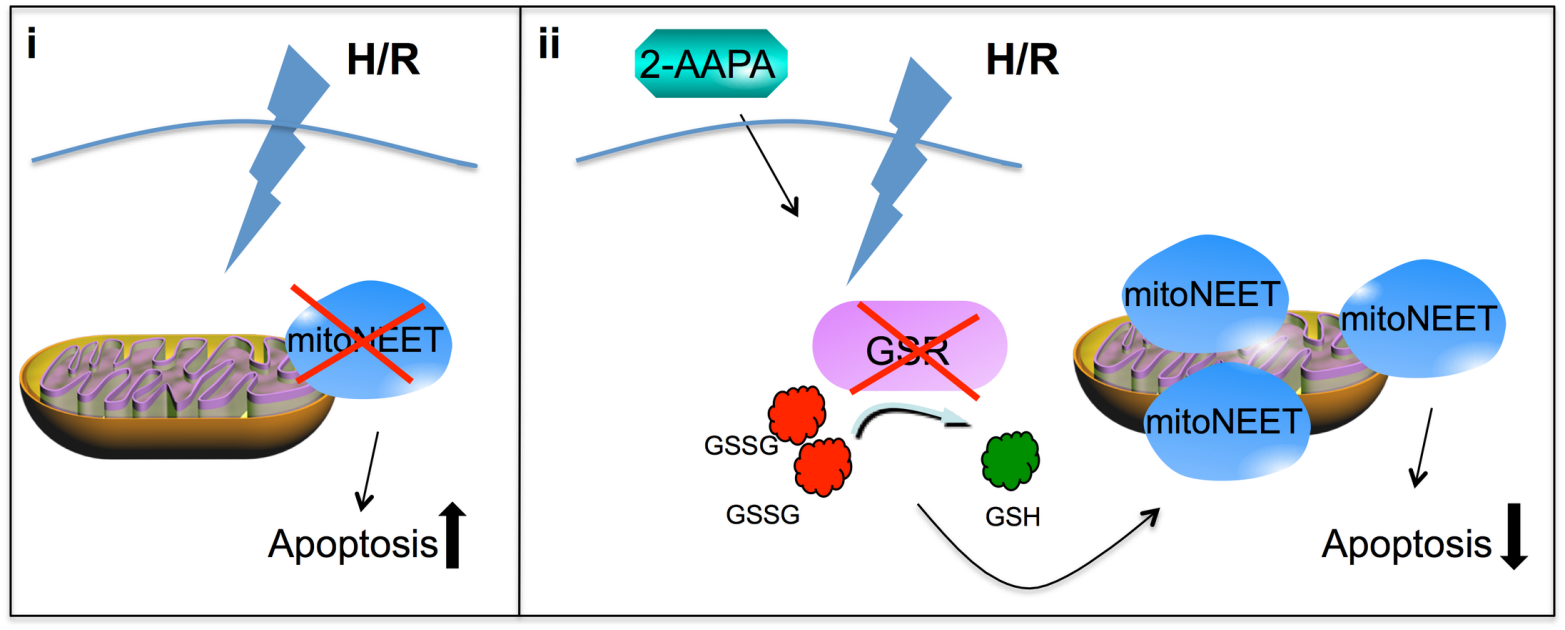
Schematic view. (i) MitoNEET-knockdown increases oxidative stress induced apoptosis. (ii) 2-AAPA inhibits GSR activity and diminishes the GSH/GSSG ratio resulting in decreasesd H/R induced apoptosis and an increase in mitoNEET-protein.

The salient findings of this study are that (i) mitoNEET is an antiapoptotic protein, which protects cardiomyoblasts (HL-1 cells) from oxidative stress induced apoptosis during H/R and (ii) that inhibition of GSR-activity by 2-AAPA increases the amount of mitoNEET-protein and decreases apoptosis, while addition of GSH abolishes 2-AAPA induced mitoNEET increase (Fig 7). These results suggest that mitoNEET may partly compensate for imbalances in the antioxidative glutathione-system during oxidative stress conditions.

Several studies investigated the impact of mitoNEET in disease models, which are associated with oxidative stress like obesity [13], cancer [14] and inflammation-induced Parkinson`s disease [15]. Recently the novel thiazolidinedione mitoNEET ligand-1 has been reported to show a protective effect on cardiac stem cell survival under oxidative stress conditions [35]. However, the role of mitoNEET during myocardial ischemia and reperfusion injury, which is also related to increased oxidative stress has not been characterized yet.

In cardiovascular diseases generation of ROS is a critical factor [36, 37]. Counteracting oxidative stress with different antioxidants diminished injury and improved recovery from ischemia and reperfusion injury in laboratory studies [4, 38] but most attempts to carry this strategy into the clinical arena have failed or demonstrated conflicting results [4].

In order to better understand the functional role of mitoNEET in the context of ischemia and reperfusion we used the *in vitro* model of H/R induced apoptosis and show that reduced mitoNEET content increased apoptosis of cardiac HL-1 cells after H/R without showing an effect on necrosis supporting the critical role of mitoNEET as an antiapoptotic but not antinecrotic protein.

To analyze how oxidative stress dependent apoptosis is affected by mitoNEET we used the two different antioxidative compounds, Tiron and GSH-MEE [39, 40]. Protecting cells from superoxide by Tiron decreased apoptotic cell death and unmasked a mitoNEET-independent effect on apoptosis as well, suggesting that additional antioxidative systems are crucial in reducing ROS-related apoptosis in cardiomyocytes. Replenishing the intracellular pool of GSH by GSH-MEE, thereby protecting cells from ROS such as hydrogen peroxide and peroxynitrite [41], minimized apoptosis to the normoxic control level. These data indicate that mitoNEET constitutes a relevant part of the antioxidative capacity of the cardiomyocyte. Indeed, in mouse models of obesity, overexpression of mitoNEET in adipocytes and hepatocytes showed decreased ROS-induced damage, whereas a systemically induced knockdown of mitoNEET expression caused the opposite effect, [13] confirming the importance of mitoNEET in several disease states associated with oxidative stress. To assess the impact of mitoNEET on ROS homeostasis we measured intracellular ROS in our H/R-model. Recent studies showed that in embryonic fibroblasts lacking the 2Fe-2S-cluster containing NEET protein Miner1, also known as NAF-1, ROS-production was increased [42]. To our surprise, we couldn’t detect a mitoNEET dependent effect on intracellular ROS production in our H/R-model. We do not know whether these differences between mitoNEET and NAF-1 are related to differences in the individual protein function or to differences in the *in vitro* cell culture models. Nevertheless, our data indicate that the presence of mitoNEET is important for mediating ROS-dependent apoptosis but it is not regulating ROS itself. It is well possible that mitoNEET interacts with other anti-apoptotic proteins or regulates the activity of anti-apoptotic enzymes. In this context it is of interest that NAF-1 was found to directly interact with Bcl-2 and Beclin 1 and therefore was proposed to regulate autophagy and apoptosis [43].

As a result of oxidative stress, reduced glutathione (GSH) is converted into its oxidized and antioxidative inactive form glutathione disulfide (GSSG). Reduced glutathione is reconstituted by GSR [18]. Several studies document decreased GSR-activity in animal models of myocardial infarction [30], ischemia and reperfusion [28] and in human patients with infarcted and reperfused hearts [29] which directed us to examine the interaction of GSR as a key enzyme of the glutathione redox system with mitoNEET-fostered anti-apoptotic effects. In our model of H/R GSR-activity was reduced (data not shown) suggesting that HL-1 cells constituted a good model for the *in vivo* situation. In order to investigate the influence of GSR-enzyme activity on H/R-dependent apoptosis we used 2-AAPA as a potent pharmacological inhibitior of the enzyme [31, 32, 34]. Reduction of GSR-activity to 20% by 2-AAPA surprisingly caused an antiapoptotic effect suggesting that in case of an extreme imbalance of the glutathione redox-system a compensatory mechanism is induced. The individual regulation of the antioxidant capacity of different antioxidative enzymes and their mutual interactions are not fully understood. It is however conceivable that one antioxidant may compensate for another to maintain the cellular redox potential [44]. Interestingly, inhibition of GSR-activity by 2-AAPA increased mitoNEET-protein dramatically, and consequently apoptosis was significantly reduced. This increase of mitoNEET-protein is most likely due to regulatory processes on the protein level since we couldn’t detect any major changes in mRNA expression of mitoNEET neither induced by H/R nor by 2-AAPA. To test, whether elevated mitoNEET-protein levels are directly caused by inhibition of GSH-restoring GSR-activity, we replenished intracellular GSH pool by addition of GSH-MEE, thereby avoiding 2-AAPA induced GSR-blockade. MitoNEET increase was completely abrogated by addition of GSH-MEE indicating that changes in glutathione availability can reverse the effect on mitoNEET-protein. In the same context a recent report by Laundry et. al. (2015) [45] showed that mitoNEET can be directly reduced by GSR in an NADPH-dependent reaction and further proposed a model for GSR-dependent redox transition of mitoNEET in response to oxidative stress. Our data support this model and add the finding that mitoNEET content is regulated by the redox state of the glutathione system suggesting a compensatory mechanism to protect cells from oxidative stress.

Nevertheless, the underlying mechanism by which decreased GSH-recycling GSR-activity increases mitoNEET content is unknown so far. 2-AAPA is a novel membrane-permeable compound that inhibits GSR-activity effectively by irreversible thiocarbamylation of GSR’s active site thiols [31]. The inhibitor does not impair the antioxidative enzymes catalase and superoxide dismutase, the enzymes responsible for GSH biosynthesis γ-glutamylcysteine synthetase and glutathione synthase and only marginally influences glutathione peroxidase and glutathione S-transferase [31, 34]. In agreement, we and others show no influence on GSR-protein and -mRNA expression [34] by 2-AAPA, respectively. Since GSR catalyzes the reduction of GSSG to GSH [44], inhibition of GSR leads to GSSG accumulation and increases intracellular thiol oxidative stress [31]. However, Zhao et al. detected no changes in ROS formation during GSR inhibition by 2-AAPA [34]. During irradiation with generation of free radicals [46], 2-AAPA-mediated inhibition of GSR aggravated thiol oxidative stress and enhanced sensitivity of cancer cells to radiation [33]. Recently, it was shown that 2-AAPA is also able to inhibit GSR-related enzyme glutaredoxin-1 [47] and thioredoxin reductase [32] suggesting that in addition to GSR blockade a possible reduction of glutaredoxin-1- and thioredoxin-activity could additionally enlarge oxidative stress during H/R.

Our study is limited by the fact that a cardiomyocyte cell line was used. Therefore we cannot conclude that our results will also be valid in an *in vivo* model. Furthermore mitoNEET-knockdown was incomplete. Since there is no bioassay for mitoNEET available we were unable to directly measure mitoNEET antioxidative activity. Regulation of enzyme activity is therefore also an aspect that could be responsible for the observation presented here. Our attempt to visualize changes in the subcellular distribution of mitoNEET were limited by the resolution of our fluorescence imaging system in combination with H/R induced morphological alterations. To make a clear statement whether mitoNEET might move intracellularly after H/R and/or treatment with 2-AAPA using electron microscopy would be more appropiate.

In conclusion we show that mitoNEET protects intact cardiac cells from oxidative stress induced apoptosis during hypoxia and reoxygenation. Additionally our study demonstrates a previously unrecognized effect of the GSR inhibitor 2-AAPA, which increases mitoNEET-protein thereby setting off the prooxidative effects of GSR-inhibition. However, further studies are needed to clarify the mechanism by which mitoNEET is elevated as well as the resulting impact on cell viability *in vivo*.

## Supporting Information

S1 Data Original data sets. (XLSX)

S1 Fig Evaluation of polyclonal anti-mitoNEET antibody directed against peptide EP083398 in rabbit SY1094. (A) Knockdown (KD) of mitoNEET by small interfering RNA (siNRA) transfection diminished mitoNEET-protein detection in HL-1 cells as demonstrated by a representative Western Blot. (B) Incubation of murine fEnd.5 protein with mitoNEET-peptide EP083398 (ratio 1:5) showed reduced detection of mitoNEET-protein by Western Blot. (C) fEnd.5 cells were stained with mitochondrion-selective dye MitoTracker (100 nM) and incubated with rabbit anti-mitoNEET antibody (1:500) and fluorescein isothiocyanate-conjugated rabbit IgG (1:50) as secondary antibody. Nuclei were tagged with 1 μg/μl DAPI. Cell stainings were visualized by an inverted microscope (Olympus IX81) using a 60x objective together with a 1.6x magnification changer and photographed by a fluorescence camera (Retiga EXi). Characteristic fluorescent stainings of nuclei (blue), mitoNEET (green), MitoTracker (red) and colocalization of mitoNEET with MitoTracker in an Overlay (orange/ yellow) are shown.
(TIFF)

S2 Fig MitoNEET expression of HL-1 cells after siRNA mediated mitoNEET-KD. (A-B) MitoNEET-mRNA (n = 12) and -protein (n = 14) amounts were reduced to 25% after mitoNEET-KD and hypoxia and reoxygenation (H/R) measured by real-time RT-PCR and Western Blot, respectively. A representative Western Blot is shown. Data were analyzed densitometrically, normalized to housekeeping gene expression and expressed as % of H/R control. Real-time RT-PCR signals were normalized to hypoxanthine phospho-ribosyltransferase (HPRT) gene expression and data are expressed as 2^-ΔΔCT^.
(TIFF)

S3 Fig Overexpression of mitoNEET reduces H/R induced apoptotic cell death. Control- and mitoNEET overexpressing-HL-1 cells were exposed to H/R. Apoptosis was detected and measured using the Cell-APOPercentage apoptosis assay (Biocolor, Tebu-bio, Offenbach, Germany) which uses a dye that is selectively imported by cells that are undergoing apoptosis. Shown are pictures of the labeled cells and the quantificiation of intracellular dye using a colorimetric assay (n = 5). Absorbance at 550 nm is measured in relative units.
(TIFF)

S4 Fig GSR expression in HL-1 cells after inhibition with 2-AAPA. GSR-protein was not affected by application of chemical GSR-inhibitor 2-AAPA (10 μM, n = 4–5). DMSO as solvent showed no influence on GSR-protein. Representative Western Blot is shown and data are expressed as % of H/R control.
(TIFF)

S5 Fig Localization of mitoNEET after treatment with 2-AAPA and GSH-MEE. HL-1 cells were treated with normoxia, H/R, H/R + 2-AAPA (10 μM) and H/R + 2-AAPA (10 μM) + GSH-MEE (2 μM). After treatment cells were stained with mitochondrion-selective dye MitoTracker (100 nM) and incubated with rabbit anti-mitoNEET antibody (1:500) and fluorescein isothiocyanate-conjugated rabbit IgG (1:50) as secondary antibody. Nuclei were tagged with 1 μg/μl DAPI. Cell stainings were visualized by an inverted microscope (Olympus IX81) using a 60x objective together with a 1.6x magnification changer and photographed by a fluorescence camera (Retiga EXi). Characteristic fluorescent stainings of nuclei (blue), mitoNEET (green), MitoTracker (red) and colocalization of mitoNEET with MitoTracker in an Overlay (orange/ yellow) are shown.
(TIFF)

S1 Protocol Evaluation of polyclonal anti-mitoNEET antibody generated against peptide EP083398 in rabbit SY1094. **Peptide competition**
Lyophilized peptide EP083398 corresponding to mouse mitoNEET-protein (UniProt ID: Q91WS0) was dissolved in 10% DMSO and combined with affinity purified polyclonal antibody (generated against peptide EP083398) in a 5-fold excess. Antigen-antibody solution was mixed by agitation (500 rpm) for 2 hours at room temperature. After centrifugation supernatant was diluted in 5% nonfat dry milk in Tris buffered saline supplemented with 0.05% Tween20 and used for incubation of protein lysates of fEnd.5 cells, a polyomavirus middle T antigen transformed murine endothelial cell line. Lysates were separated by SDS-PAGE and blotted on PVDF membranes. Protein was detected as described in Materials and Methods.
**Mitochondrial localization**
Immortalized mouse fEnd.5 cells [1,2] were plated on glass cover slips and cultured in Dulbecco`s modified Eagle`s medium supplemented with 10% fetal bovine serum, 1% L-glutamine and 1% penicillin/streptomycin until confluence. Cells were washed with PBS and incubated with 100 nM MitoTracker Orange CMTMRos (Invitrogen, Darmstadt, Germany) for 30 minutes at 37°C and prepared for indirect immunofluorescence staining as described previously [3] with some modifications. After fixation and quenching, cells were permeabilized with 0.5% saponin (Applichem, Darmstadt, Germany) in PBS for 10 minutes and washed twice with 0.1% saponin. Unspecific binding was blocked (5% normal goat serum, 0.1% saponin, 1% BSA in PBS) for 30 minutes and cells were washed three times with 1% BSA in PBS before incubation with anti-mitoNEET antibody (diluted 1:500 in 1% BSA in PBS) over night at 4°C in a wet chamber. Cells were washed 3 times with 0.1% saponin, 1% BSA in PBS. A fluorescein isothiocyanate-conjugated donkey anti-rabbit IgG antibody (Jackson ImmunoResearch, Suffolk, UK) was used as secondary antibody (diluted 1:50 in 0.1% saponin, 1% BSA in PBS) and DNA was colored blue with 1 μg/ml DAPI. Fluorescent stainings were visualised by an inverted microscope (Olympus IX81, Olympus, Muenster, Germany) using a 60x objective and a 1.6x magnification changer. Photos were taken by fluorescence camera Retiga EXi (QImaging, Buckinghamshire, United Kingdom).
Protocol apoptosis. (ii) 2-AAPA inhibits GSR activity and diminishes the GSH/GSSG ratio resulting in decreasesd H/R induced apoptosis and an increase in mitoNEET-protein.
(DOCX)

## Abbreviations

2-AAPA: 2-acetylamino-3-[4-(2-acetylamino-2-carboxyethylsulfanylthio-carbonylamino) phenylthiocarbamoylsulfanyl] propionic acid
DCF-DA: 2’,7’-dichlorofluorescin diacetate
GAPDH: glyceraldehyde-3-phosphate dehydrogenase
GSH: glutathione
GSH/GSSG: reduced glutathione to oxidized glutathione
GSH-MEE: glutathione compound glutathione reduced ethyl ester
GSR: glutathione reductase
HL-1 cells: murine cardiomyocyte cell line
HPRT: hypoxanthine phosphoribosyltransferase
H/R: hypoxia and reoxygenation
KD: knockdown
LDH: lactate dehydrogenase
ROS: reactive oxygen species
scr-siRNA: non-specific small interfering RNA
siRNA: small interfering RNA
