# Supplementary figures and images for: MitoNEET Protects HL-1 Cardiomyocytes from Oxidative Stress Mediated Apoptosis in an In Vitro Model of Hypoxia and Reoxygenation
Source: PLoS One. 2016 May 31;11(5):e0156054. doi: 10.1371/journal.pone.0156054 (PMC4887087; doi:10.1371/journal.pone.0156054)

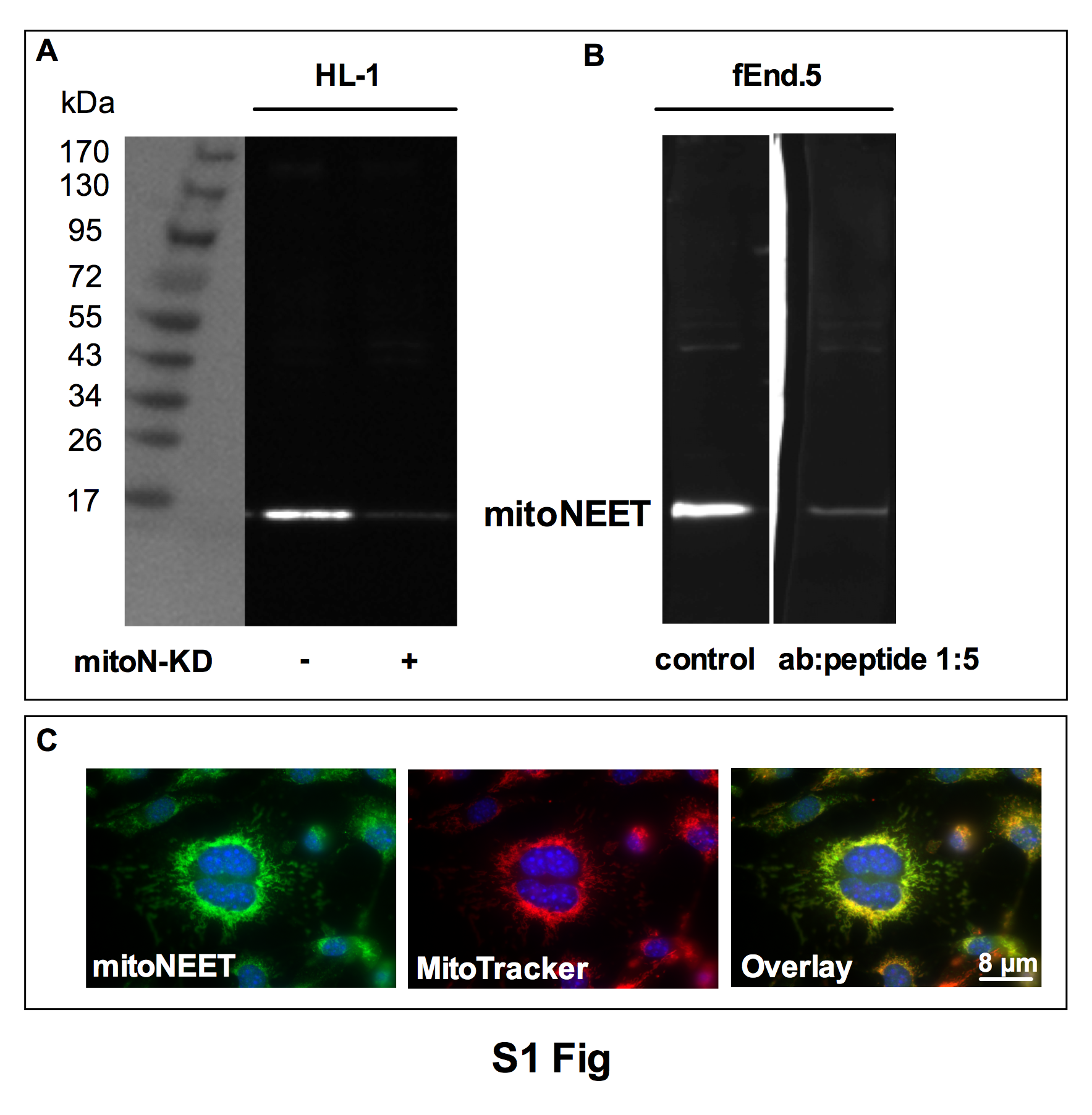

Supplement: S1 Fig — (A) Knockdown (KD) of mitoNEET by small interfering RNA (siNRA) transfection diminished mitoNEET-protein detection in HL-1 cells as demonstrated by a representative Western Blot. (B) Incubation of murine fEnd.5 protein with mitoNEET-peptide EP083398 (ratio 1:5) showed reduced detection of mitoNEET-protein by Western Blot. (C) fEnd.5 cells were stained with mitochondrion-selective dye MitoTracker (100 nM) and incubated with rabbit anti-mitoNEET antibody (1:500) and fluorescein isothiocyanate-conjugated rabbit IgG (1:50) as secondary antibody. Nuclei were tagged with 1 μg/μl DAPI. Cell stainings were visualized by an inverted microscope (Olympus IX81) using a 60x objective together with a 1.6x magnification changer and photographed by a fluorescence camera (Retiga EXi). Characteristic fluorescent stainings of nuclei (blue), mitoNEET (green), MitoTracker (red) and colocalization of mitoNEET with MitoTracker in an Overlay (orange/ yellow) are shown. (TIFF) [file pone.0156054.s002.tiff]

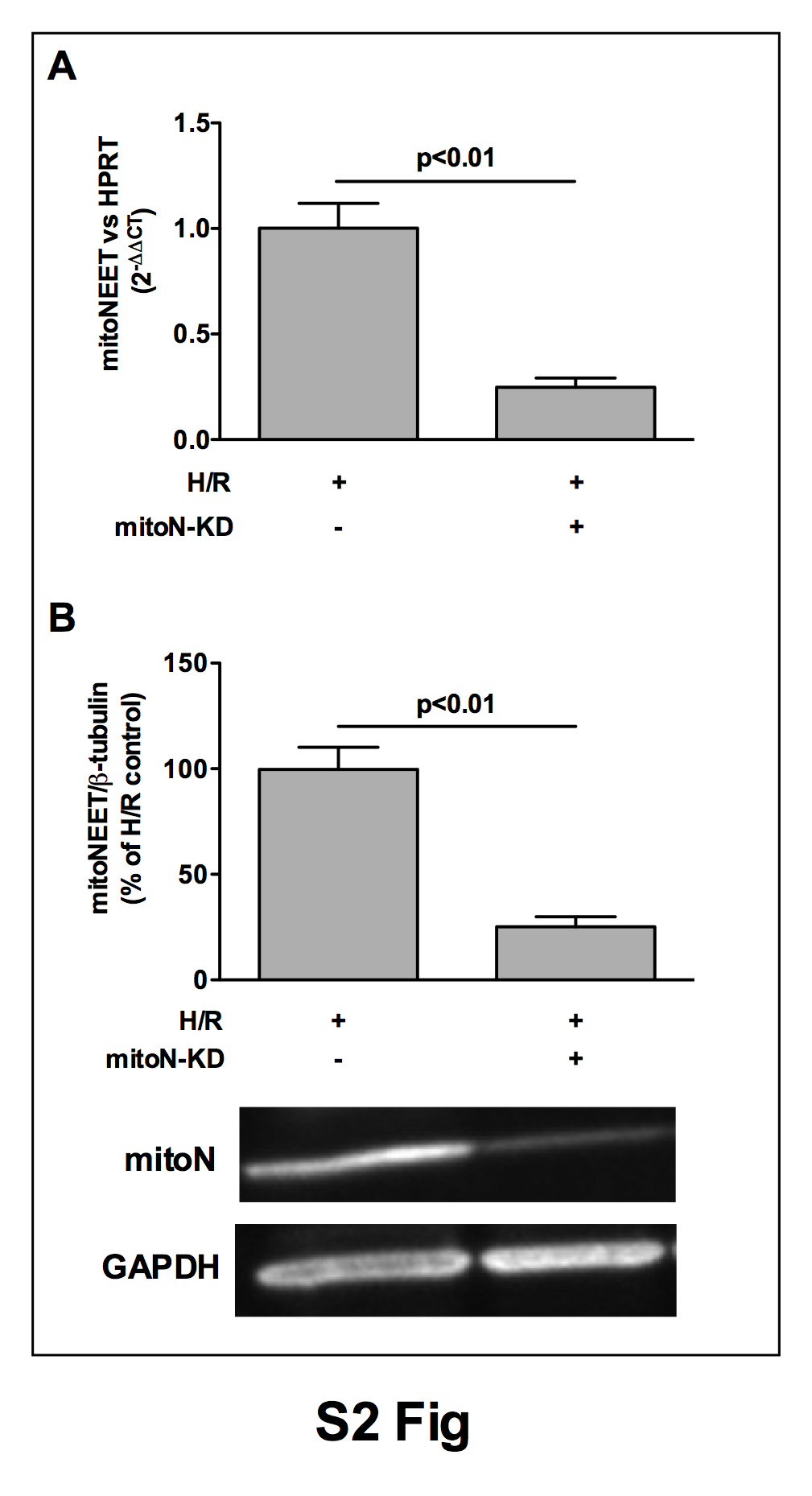

Supplement: S2 Fig — (A-B) MitoNEET-mRNA (n = 12) and -protein (n = 14) amounts were reduced to 25% after mitoNEET-KD and hypoxia and reoxygenation (H/R) measured by real-time RT-PCR and Western Blot, respectively. A representative Western Blot is shown. Data were analyzed densitometrically, normalized to housekeeping gene expression and expressed as % of H/R control. Real-time RT-PCR signals were normalized to hypoxanthine phospho-ribosyltransferase (HPRT) gene expression and data are expressed as 2-ΔΔCT. (TIFF) [file pone.0156054.s003.tiff]

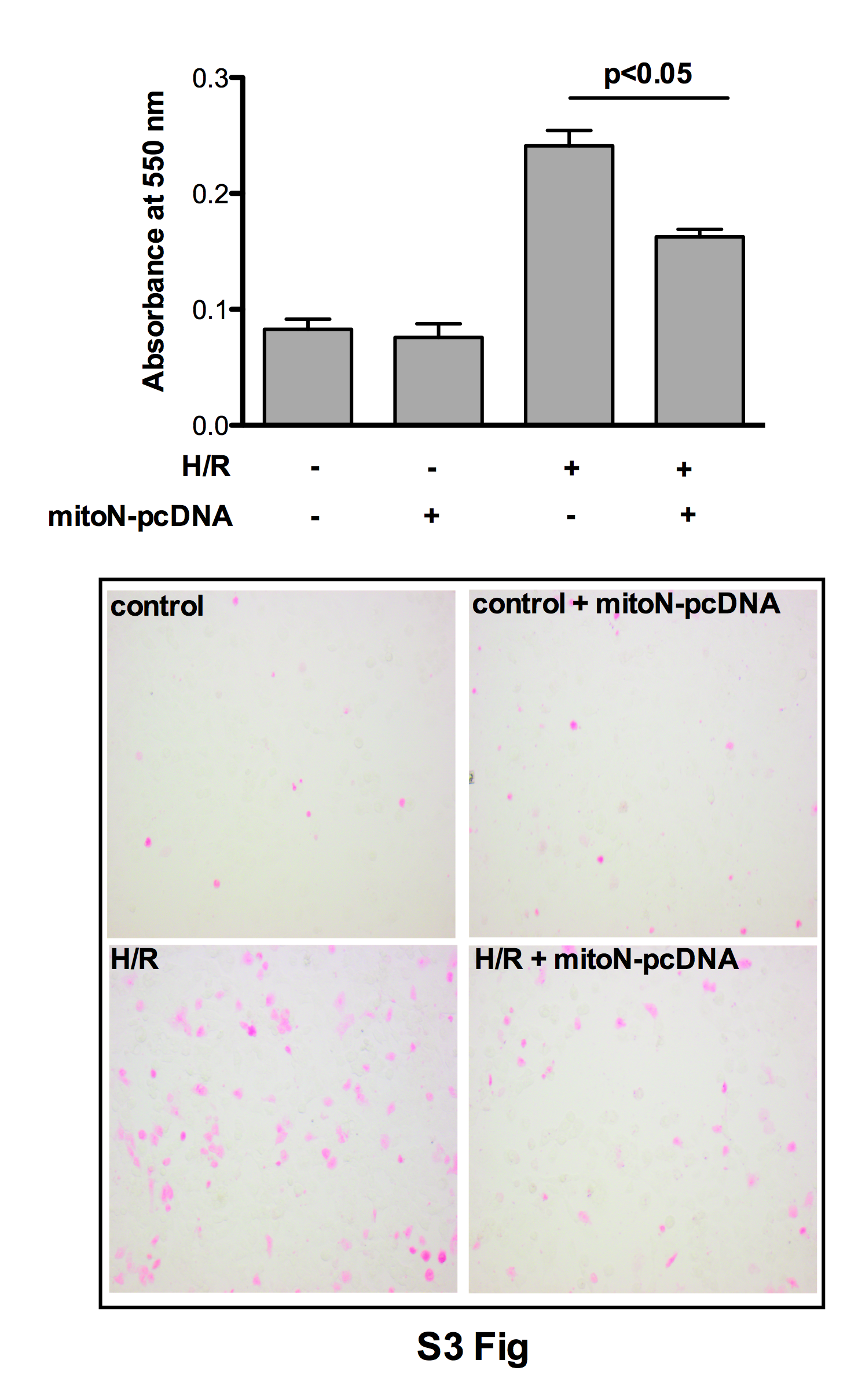

Supplement: S3 Fig — Control- and mitoNEET overexpressing-HL-1 cells were exposed to H/R. Apoptosis was detected and measured using the Cell-APOPercentage apoptosis assay (Biocolor, Tebu-bio, Offenbach, Germany) which uses a dye that is selectively imported by cells that are undergoing apoptosis. Shown are pictures of the labeled cells and the quantificiation of intracellular dye using a colorimetric assay (n = 5). Absorbance at 550 nm is measured in relative units. (TIFF) [file pone.0156054.s004.tiff]

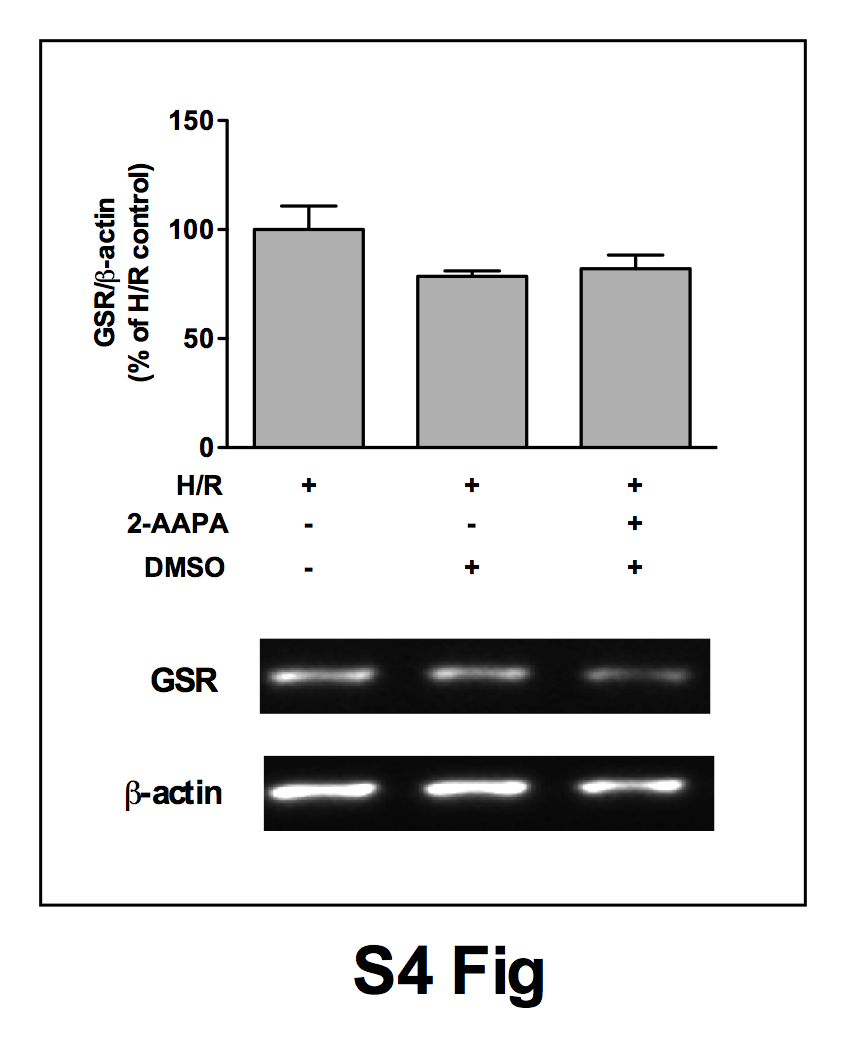

Supplement: S4 Fig — GSR-protein was not affected by application of chemical GSR-inhibitor 2-AAPA (10 μM, n = 4–5). DMSO as solvent showed no influence on GSR-protein. Representative Western Blot is shown and data are expressed as % of H/R control. (TIFF) [file pone.0156054.s005.tiff]

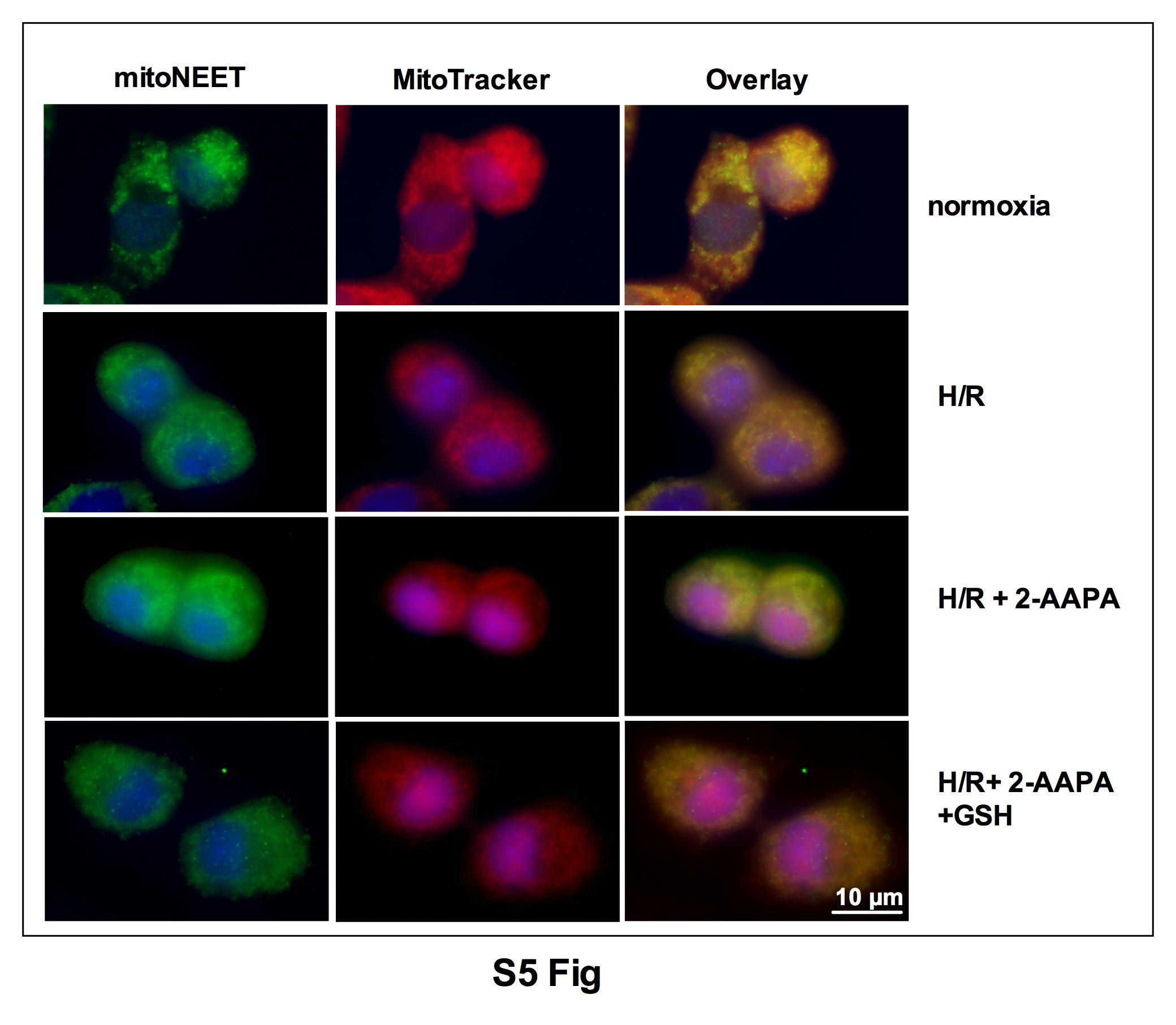

Supplement: S5 Fig — HL-1 cells were treated with normoxia, H/R, H/R + 2-AAPA (10 μM) and H/R + 2-AAPA (10 μM) + GSH-MEE (2 μM). After treatment cells were stained with mitochondrion-selective dye MitoTracker (100 nM) and incubated with rabbit anti-mitoNEET antibody (1:500) and fluorescein isothiocyanate-conjugated rabbit IgG (1:50) as secondary antibody. Nuclei were tagged with 1 μg/μl DAPI. Cell stainings were visualized by an inverted microscope (Olympus IX81) using a 60x objective together with a 1.6x magnification changer and photographed by a fluorescence camera (Retiga EXi). Characteristic fluorescent stainings of nuclei (blue), mitoNEET (green), MitoTracker (red) and colocalization of mitoNEET with MitoTracker in an Overlay (orange/ yellow) are shown. (TIFF) [file pone.0156054.s006.tiff]
