# Supplementary material for: MitoNEET Protects HL-1 Cardiomyocytes from Oxidative Stress Mediated Apoptosis in an In Vitro Model of Hypoxia and Reoxygenation
Source: PLoS One. 2016 May 31;11(5):e0156054. doi: 10.1371/journal.pone.0156054 (PMC4887087; doi:10.1371/journal.pone.0156054)
Supplement: S1 Protocol — Peptide competition Lyophilized peptide EP083398 corresponding to mouse mitoNEET-protein (UniProt ID: Q91WS0) was dissolved in 10% DMSO and combined with affinity purified polyclonal antibody (generated against peptide EP083398) in a 5-fold excess. Antigen-antibody solution was mixed by agitation (500 rpm) for 2 hours at room temperature. After centrifugation supernatant was diluted in 5% nonfat dry milk in Tris buffered saline supplemented with 0.05% Tween20 and used for incubation of protein lysates of fEnd.5 cells, a polyomavirus middle T antigen transformed murine endothelial cell line. Lysates were separated by SDS-PAGE and blotted on PVDF membranes. Protein was detected as described in Materials and Methods. Mitochondrial localization Immortalized mouse fEnd.5 cells [1,2] were plated on glass cover slips and cultured in Dulbecco`s modified Eagle`s medium supplemented with 10% fetal bovine serum, 1% L-glutamine and 1% penicillin/streptomycin until confluence. Cells were washed with PBS and incubated with 100 nM MitoTracker Orange CMTMRos (Invitrogen, Darmstadt, Germany) for 30 minutes at 37°C and prepared for indirect immunofluorescence staining as described previously [3] with some modifications. After fixation and quenching, cells were permeabilized with 0.5% saponin (Applichem, Darmstadt, Germany) in PBS for 10 minutes and washed twice with 0.1% saponin. Unspecific binding was blocked (5% normal goat serum, 0.1% saponin, 1% BSA in PBS) for 30 minutes and cells were washed three times with 1% BSA in PBS before incubation with anti-mitoNEET antibody (diluted 1:500 in 1% BSA in PBS) over night at 4°C in a wet chamber. Cells were washed 3 times with 0.1% saponin, 1% BSA in PBS. A fluorescein isothiocyanate-conjugated donkey anti-rabbit IgG antibody (Jackson ImmunoResearch, Suffolk, UK) was used as secondary antibody (diluted 1:50 in 0.1% saponin, 1% BSA in PBS) and DNA was colored blue with 1 μg/ml DAPI. Fluorescent stainings were visualised by an inverted micro [file pone.0156054.s007.docx]

**S1 Protocol: Evaluation of polyclonal anti-mitoNEET antibody generated against peptide EP083398 in rabbit SY1094.**

**Peptide competition**

Lyophilized peptide EP083398 corresponding to mouse mitoNEET-protein (UniProt ID: Q91WS0) was dissolved in 10% DMSO and combined with affinity purified polyclonal antibody (generated against peptide EP083398) in a 5-fold excess. Antigen-antibody solution was mixed by agitation (500 rpm) for 2 hours at room temperature. After centrifugation supernatant was diluted in 5% nonfat dry milk in Tris buffered saline supplemented with 0.05% Tween20 and used for incubation of protein lysates of fEnd.5 cells, a polyomavirus middle T antigen transformed murine endothelial cell line [1,2]. Lysates were separated by SDS-PAGE and blotted on PVDF membranes. Protein was detected as described in Materials and Methods.

**Mitochondrial localization**

Immortalized mouse fEnd.5 cells [1,2] were plated on glass cover slips and cultured in Dulbecco`s modified Eagle`s medium supplemented with 10% fetal bovine serum, 1% L-glutamine and 1% penicillin/streptomycin until confluence. Cells were washed with PBS and incubated with 100 nM MitoTracker Orange CMTMRos (Invitrogen, Darmstadt, Germany) for 30 minutes at 37ºC and prepared for indirect immunofluorescence staining as described previously [3] with some modifications. After fixation and quenching, cells were permeabilized with 0.5% saponin (Applichem, Darmstadt, Germany) in PBS for 10 minutes and washed twice with 0.1% saponin. Unspecific binding was blocked (5% normal goat serum, 0.1% saponin, 1% BSA in PBS) for 30 minutes and cells were washed three times with 1% BSA in PBS before incubation with anti-mitoNEET antibody (diluted 1:500 in 1% BSA in PBS) over night at 4ºC in a wet chamber. Cells were washed 3 times with 0.1% saponin, 1% BSA in PBS. A fluorescein isothiocyanate-conjugated donkey anti-rabbit IgG antibody (Jackson ImmunoResearch, Suffolk, UK) was used as secondary antibody (diluted 1:50 in 0.1% saponin, 1% BSA in PBS) and DNA was colored blue with 1 µg/ml DAPI. Fluorescent stainings were visualised by an inverted microscope (Olympus IX81, Olympus, Muenster, Germany) using a 60x objective and a 1.6x magnification changer. Photos were taken by fluorescence camera Retiga EXi (QImaging, Buckinghamshire, United Kingdom).

**References**

1. Theilmeier G, De Geest B, Van Veldhoven PP, Stengel D, Michiels C, Lox M, et al. HDL-associated PAF-AH reduces endothelial adhesiveness in apoE-/- mice. FASEB journal : official publication of the Federation of American Societies for Experimental Biology. 2000;14(13):2032-9. doi: 10.1096/fj.99-1029com. PubMed PMID: 11023987.

2. Conway EM, Van de Wouwer M, Pollefeyt S, Jurk K, Van Aken H, De Vriese A, et al. The lectin-like domain of thrombomodulin confers protection from neutrophil-mediated tissue damage by suppressing adhesion molecule expression via nuclear factor kappaB and mitogen-activated protein kinase pathways. The Journal of experimental medicine. 2002;196(5):565-77. PubMed PMID: 12208873; PubMed Central PMCID: PMC2193995.
